# Structure-Based High-Throughput Epitope Analysis of Hexon Proteins in B and C Species Human Adenoviruses (HAdVs)

**DOI:** 10.1371/journal.pone.0032938

**Published:** 2012-03-13

**Authors:** Xiao-Hui Yuan, Ying-Chen Wang, Wen-Jing Jin, Bin-Bin Zhao, Cai-Feng Chen, Jian Yang, Jing-Fei Wang, Ying-Ying Guo, Jing-Jun Liu, Ding Zhang, Lu-Lu Gong, You-Wen He

**Affiliations:** 1 Key Laboratory of Systems Biology of Pathogens, Ministry of Health, The Institute of Pathogen Biology, Chinese Academy of Medical Sciences & Peking Union Medical College, Beijing, China; 2 Department of Hygienic Microbiology, School of Public Health, Harbin Medical University, Harbin, Heilongjiang, China; 3 Harbin Veterinary Research Institute, Chinese Academy of Agricultural Sciences, Harbin, Heilongjiang, China; 4 Department of Immunology, Duke University Medical Center, Durham, North Carolina, United States of America; French National Centre for Scientific Research, France

## Abstract

Human adenoviruses (HAdVs) are the etiologic agent of many human infectious diseases. The existence of at least 54 different serotypes of HAdVs has resulted in difficulties in clinical diagnosis. Acute respiratory tract disease (ARD) caused by some serotypes from B and C species is particularly serious. Hexon, the main coat protein of HAdV, contains the major serotype-specific B cell epitopes; however, few studies have addressed epitope mapping in most HAdV serotypes. In this study, we utilized a novel and rapid method for the modeling of homologous proteins based on the phylogenetic tree of protein families and built three-dimensional (3D) models of hexon proteins in B and C species HAdVs. Based on refined hexon structures, we used reverse evolutionary trace (RET) bioinformatics analysis combined with a specially designed hexon epitope screening algorithm to achieve high-throughput epitope mapping of all 13 hexon proteins in B and C species HAdVs. This study has demonstrated that all of the epitopes from the 13 hexon proteins are located in the proteins' tower regions; however, the exact number, location, and size of the epitopes differ among the HAdV serotypes.

## Introduction

HAdVs are nonenveloped, double-stranded DNA (dsDNA) viruses with icosahedral capsids [1]. At least 54 standard HAdV serotypes are presently recognized based mainly on the neutralization by specific antisera. These serotypes are in turn divided into six species (A, B, C, D, E, and F, with B species further divided into B1 and B2 sub-species) [2], [3], [4]. HAdVs are associated with a wide spectrum of human clinical infectious diseases [1], [5], [6], [7], including ARD [8], conjunctivitis, viral gastroenteritis, acute haemorrhagic cystitis, encephalitis, and obesity, and seriously threaten human health. It is generally believed that HAdV-related ARD is mainly caused by the B1 (B3, B7, B16, B21, and B50) and C (C1, C2, C5 and C6) species HAdVs [9], [10], [11], [12]; however, recent studies indicate that B2 (B11, B14, B34, and B35) species HAdVs also cause serious respiratory infections and some outbreaks [13]. The existence of at least 13 different serotypes of B and C species HAdVs and co-infection has brought difficulties to the clinical diagnosis [14].

Hexon homotrimer, the main coat protein of HAdV, contains many serotype-specific B cell epitopes in its tower region [15]. Although the identification of hexon epitopes is essential for the production of rapid diagnostic reagents for HAdVs, few studies have addressed the precise epitope mapping of most HAdV hexon proteins. The traditional bioinformatics epitope prediction methods such as hydrophilicity, secondary structure, and flexibility analysis are all based on the primary protein sequences [16], [17], [18], but these approaches have inherent blindness. Some reports have shown that two loops (Loop1 and Loop2) on the hexon protein containing seven hypervariable regions (HVRs) may contain the epitopes [19], [20], [21]; however, the HVRs do not directly correspond to the serotype-specific B cell epitopes and cannot be directly used to develop rapid adenovirus diagnostic reagents [22], [23].

Because hexon is a protein homotrimer, analysis of the 3D structure of this complex is necessary to obtain accurate epitope information; however, only the structures of serotype 2 and 5 HAdV hexons have been determined by X-ray crystal diffraction [24], [25]. In our previous study, we used the homology modeling method to determine the 3D structure of human adenovirus serotype 3 (HAdV3) hexon and predicted its epitopes using this method in combination with a bioinformatics epitope screening algorithm based on the two important features of hexon type-specific B cell epitopes. These putative epitopes have been successfully validated by ELISA and neutralization tests (NTs) [26], demonstrating the reliability of this structure-based epitope screening method.

With the rapid development of high-throughput genome sequencing technology [14], increasing numbers of HAdV hexon family proteins have been sequenced. Rapid structure modeling of hexon protein family members is essential for high-throughput hexon epitope mapping and the development of diagnostic reagents for adenovirus typing. Although the homology modeling approach has the capacity of calculating the structure of a single unknown molecule [27], [28], [29], it requires a long cycling time and is cumbersome when working with multiple molecules of the hexon family; thus, it cannot achieve a rapid structural modeling and structure-based epitope mapping of hexon proteins. Moreover, each adenovirus hexon homotrimer contains approximately 2,800 amino acid residues, and the initial structure obtained from homology modeling must be refined by molecular mechanics (MM) energy minimization and long-range molecular dynamics (MD) simulation in an explicit solvent environment [28]. Because performing separate modeling and refining processes requires a large amount of time and computing resources, it is necessary to establish a rapid and accurate modeling method for structural analysis of multiple members of a protein family, such as adenovirus hexon proteins, to provide a basis for further rapid high-throughput mapping of serotype-specific B cell epitopes.

The “first principle of homology modeling” [28], [30], [31] states that the homology value, which represents the sequence shared by the target sequence and the template, directly determines the accuracy of modeling and the workload. A higher homology value indicates a more accurate modeling structure and less calculation to refine the initial structure. In general, when the homology among sequences is more than 30%, the result of homology modeling is reliable; when the sequence homology between two proteins is higher than 50%, less than 10% of the Cα atoms in the proteins deviate by more than 1.0 Å (0.1 nm).

The homology among different sequences can be determined through phylogenetic analysis. The present study utilizes a novel rapid molecular modeling method [32] to build hexon proteins of B and C species HAdVs based on the distance information obtained from the phylogenetic tree. Using the “first principle of homology modeling,” a phylogenetic tree of hexon amino acid sequences in B and C species HAdVs was constructed, and an optimal modeling path was determined. Next, all HAdV hexon family proteins were built and refined using the Modeler [33] and Charmm [34] programs. Further bioinformatics analysis was performed based on the following two characteristics of serotype-specific B cell epitopes of HAdV hexons: 1) the amino acids of the B cell epitope should be located on the surface of the antigen molecules and 2) serotype-specific epitope sequences should be exclusive to each serotype. By combining structural properties and bioinformatics analysis, we have improved the RET method and the purposely designed hexon epitope screening algorithm used in a previous study [26] and utilized this new method to screen epitopes from the 13 HAdV hexons examined in this study. The results demonstrate that all the 3D structures of the 13 hexon proteins are reasonable and all the structures are almost the same as the results from the traditional homology modeling method (average RMSD less than 0.1 nm), but the time taken is just 1/3 of the traditional method. All the predicted epitopes are located in the tower regions; however, the number, location, size, and 3D structure of the epitopes differ among viral serotypes. These candidate epitopes can be directly used in rapid development of HAdV diagnostic serotyping reagents and have the potential to be broadly applied.

## Materials and Methods

### Sequences and multiple sequence alignment (MSA)

Amino acid sequences of B and C species HAdV hexons were obtained from GenBank database under the following accession numbers: X76549 (B3), AC_000018 (B7), AC_000015 (B11), DQ149612 (B14), AY601636 (B16), AY008279 (B21), AB052911 (B34), AB052912 (B35), DQ149643 (B50), AC_000017 (C1), 1P2Z_A (C2), 1P30_A (C5) and DQ149613 (C6). There are three adenovirus hexon proteins available in the protein database (PDB): the chimpanzee adenovirus serotype 68 hexon (AdC68 PDB_ID: 2OBE; resolution: 2.10 Å) [15], HAdV serotype 2 hexon (HAdV2 PDB_ID: 1P2Z; resolution: 2.20 Å) [24], and HAdV serotype 5 hexon (HAdV5 PDB_ID: 1P30; resolution: 2.50 Å) [25], and the latter two are the target sequences of this work. These three hexons were used as templates for structure modeling in this study. In order to determine the homology among these thirteen hexon sequences and template sequences, MSA was performed with Clustal W 1.83 software [35] using a progressive algorithm and adjusted manually. A homology matrix of 14 hexon sequences was generated as shown in Table 1.

**Table 1:** The homology matrix of fourteen hexon sequences.

	B3	B7	B11	B14	B16	B21	B34	B35	B50	C1	C2	C5	C6	2OBE
B3	100	95.4	87.0	86.5	88.2	86.8	85.8	86.6	87.2	79.8	79.6	79.7	79.4	86.6
B7	95.4	100	88.0	87.7	88.9	88.1	86.9	87.8	88.4	80.3	80.5	79.4	80.0	87.2
B11	87.0	88.0	100	92.5	87.7	93.9	92.6	96.6	91.9	78.8	79.0	78.7	78.0	86.4
B14	86.5	87.7	92.5	100	87.6	90.9	94.9	92.1	94.2	78.7	78.8	78.4	77.8	86.8
B16	88.2	88.9	87.7	87.6	100	87.4	87.2	87.3	87.8	79.9	79.8	79.8	79.5	88.3
B21	86.8	88.1	93.9	90.9	87.4	100	90.6	93.0	93.5	79.0	79.2	79.2	78.0	86.9
B34	85.8	86.9	92.6	94.9	87.2	90.6	100	92.1	93.3	78.1	78.6	78.2	77.3	86.9
B35	86.6	87.8	96.6	92.1	87.3	93.0	92.1	100	91.6	78.7	78.9	78.1	77.9	85.9
B50	87.2	88.4	91.9	94.2	87.8	93.5	93.3	91.6	100	79.3	79.6	79.3	79.0	87.9
C1	79.8	80.3	78.8	78.7	79.9	79.0	78.1	78.7	79.3	100	90.8	87.7	91.4	81.2
C2	79.6	80.5	79.0	78.8	79.8	79.2	78.6	78.9	79.6	90.8	100	87.9	90.6	80.5
C5	79.7	79.4	78.7	78.4	79.8	79.2	78.2	78.1	79.3	87.7	87.9	100	88.6	80.4
C6	79.4	80.0	78.0	77.8	79.5	78.0	77.3	77.9	79.0	91.4	90.6	88.6	100	80.1
2OBE	86.6	87.2	86.4	86.8	88.3	86.9	86.9	85.9	87.9	81.2	80.5	80.4	80.1	100

### Phylogenetic analysis and determination of modeling path

**Figure 1:**
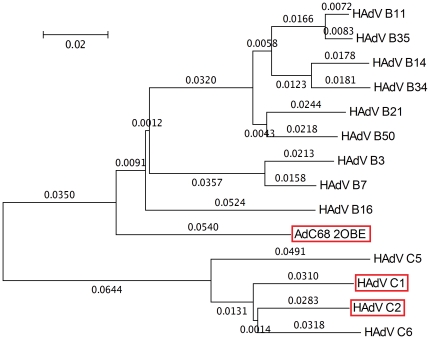
Phylogenetic tree of hexon proteins of HAdVs. The phylogenetic tree was inferred using the NJ method. The numbers in the tree show the distance between different sequences. The tree is drawn to scale with branch lengths (next to each branch) in the same units as those of the evolutionary distances used to infer the phylogenetic tree.

The phylogenetic analysis performed in this study employed all hexon protein sequences from B and C species HAdVs and the template hexon 2OBE. Based on sequences previously aligned by MSA, an unrooted phylogenetic tree reflecting the distance relationship of all hexon amino acid sequences was constructed with the MEGA 4.0 [36] package based on the Neighbor-Joining (NJ) method [37]. According to the “first principle of homology modeling,” an optimal modeling path was determined for rapid homology modeling of these hexon proteins based on the clade and distance information (clade was preferred) (Figure 1).

According to the distance information provided by the phylogenetic tree, a progressive modeling path is determined. The modeling path is divided into two parts, a branch for the B species and the other one for C. For the first B branch, 2OBE was used as the main template for modeling of the nine HAdV B species hexons, among which, B16 hexon was the one closest to 2OBE. Therefore, B16 was chosen as the starting point of the B branch modeling path and used as the template to carry out the modeling of B7. B7 was then used as the template to perform the modeling of B3 and B50; B50 was used as the template for B21 and B11; B11 was used as the template for B35 and B14; and B14 was used as the template for the modeling of B34.

For the second C branch: 1P2Z (C2) and 1P30 (C5) from PDB are used as target sequences, so they are chosen as the starting point of the C branch modeling path; 1P2Z was used as the template to carry out the modeling of C1, C2, and C6, while 1P30 was used as the template to carry out the modeling of C5. There is no downstream sequence of C5, and as a node molecule C5 is fully refined.

### Building the initial structures of B16, C2, and C5

The homology modeling of B16, C2, and C5 hexons was performed with life science software Discovery Studio 2.5 (Accelrys Software, Inc., San Diego, CA). The hexon trimer sequences and the corresponding template sequences were aligned accurately. Structurally conserved regions (SCRs) and variable regions (VRs) included in Loop 1 (100–300) and Loop 2 (400–450) were determined based on the sequence alignment. The initial 3D model of B16, C2, and C5 were built using the Modeler program and an *ab initio* loop prediction algorithm LOOPER [38] was performed to refine VR areas. All calculations for building the initial structure were performed using an SGI Vitu VS 100 graphics workstation.

### Structure refinement of B16, C2, and C5

The refinement process can be divided into two parts: MM energy minimization and explicit solvent MD balancing simulation. Both calculations were performed with the fast molecular dynamics simulation software package Gromacs [39] using charmm27 force field [40], [41]. First, the initial models was dissolved in rectangular boxes containing SPC/E (simple-point-charge) [42] water molecules, and a certain number of neutralizing Na^+^ or Cl^−^ ions were added to neutralize the negative charge. After removing bad contacts by steepest descent (SD) and conjugate gradient (CG) energy minimizations and relaxing water solvents by position-restrained MD simulations, a final 10-ns production MD simulation for each hexon was performed under periodic boundary conditions with time step of 2 fs at 310 K (∼37°C). Electrostatic interactions were calculated using the Particle Mesh Ewald (PME) [43] summation scheme. The conformations were stored every 5 ps. Finally, the MD simulation was analyzed in terms of potential energy (PE) and root-mean-square-deviation (RMSD) [44] from the initial model structure to determine whether the structures were balanced using the Gromacs suite of programs. In order to estimate the flexibility of particular hexon regions, especially the epitopes-containing amino acids on Loop 1 and 2 during the last balanced phase of MD simulation, the root-mean-square-fluctuation (RMSF) [45] of Cα was calculated simultaneously. The average structures were selected from the last stable phase and further refined to obtain the final structure. Molecular graphics images were generated using Discovery Studio software. All of the Gromacs MD simulations were performed in the DAWNING Supercomputer Center (64 Cores).

### Rapid modeling of B and C species hexons and structure assessment

According to the “first principle of homology modeling”, all other ten hexons were built progressively along the modeling path and with short refinement on the Accerys Discovery Studio 2.5 platform. The running parameters were as follows: for each hexon, the initial structure modeling was performed using the same method described for the modeling of the B16, C2, and C5 hexons, and the Charmm module of Discovery Studio was then utilized to refine each model. MM and short MD simulation were performed under charmm27 force field. The models were first dissolved in the implicit solvent environment (Generalized Born with a Simple Switching, GBSW) [46], and 2000 steps of the SD and CG methods were employed until the whole system convergence criterion reached 0.4184 kJ/(mol·nm). A 5-ps MD simulation was then performed with a system temperature ranging from 50 to 310 K, and then the total 50-ps production MD simulation was performed at 310 K. The system's long-range electrostatic interactions were calculated using the spherical cutoff method.

The thirteen final hexon structures were further checked for stereochemical accuracy and residue compatibility with the Procheck online program [47] and the Profile-3D [48] program in Discovery Studio, respectively. All of the calculations were performed on a Dell PowerEdge 2900 workstation.

### Comparison with traditional modeling

In order to test the accuracy of new modeling method and acceleration on time frame compared with the traditional homology modeling, the other ten hexon molecules were modeled by the traditional homology modeling method using the same procedure of B16, C2, and C5 modeling. Then again, a total 10-ns explicit solvent MD simulation was performed by Gromacs for each hexon in the same calculation environment. The resulting structures were superimposed with the structures from the new rapid method, and pairwise structural RMSD calculation was performed in Discovery Studio 2.5 platform.

In order to accurately test the superiority of the new modeling method over the traditional modeling method in the time frame, the authors have only considered the CPU time consumed by the two methods in the same computing environment.

### Solvent accessibility surface (SAS) analysis

SAS analysis is commonly used to evaluate how deep a given residue is buried [49]. The SAS of each hexon structure was calculated by Solvent Accessibility Calculation of Discovery Studio 2.5 software with probe radius of 1.4 Å. The differences in SAS among residues in the hexon models were determined. Two residue groups were created: 1) exposed group, which contained residues with maximum SAS values greater than 25%, and 2) buried group, which contained residues with maximum SAS less than 10%. These SAS data were used in the subsequent epitope screening.

### Reverse Evolutionary Trace analysis

RET analysis includes MSA, site homology calculation, and 3D mapping [26], [50]. In order to probe the serotype-specific epitopes of all HAdVs and the serotype-specific epitopes of B and C species HAdVs, two MSAs were performed using the Clustal X 1.83 software. For the first MSA, in addition to the thirteen hexon sequences, ten representative sequences from HAdV species A, D, E, and F in GenBank under the accession numbers X73487 (A12), DQ149610 (A18), DQ149611 (A31), NC_003266 (E4), DQ149615 (D10), DQ149617 (D15), DQ149632 (D37), EF153473 (D48), X51782 (F40), and DQ315364 (F41) were also included. For the second MSA, only B and C species internal hexon sequences were included. The site homology value, which reflects the degree to which a certain site is conserved in aligned homologous protein sequences, was then calculated according to the aligned sequences in both MSAs. This calculation was performed as described previously [26]. Briefly, site homology = number of conserved amino acids on same site/number of total sequences×100%.

### High-throughput epitope screening

An epitope screening algorithm was designed combining the data from SAS and the site homology analysis of RET according to the two features of hexon serotype-specific B cell epitopes. The algorithm was as follows. Sites with homology of less than 45% were defined as hypervariable. In the first MSA, segments fulfilling the following criteria were selected as candidates: 1) the length of the site is between 6 and 18 amino acids; 2) more than half of the sites are hypervariable; 3) the interval between candidate sequences is not shorter than three amino acids; and 4) 90% of the residues belong to the exposed group with the maximum SAS greater than 25%. The same criteria were also applied to the second screening cycle (second MSA) to refine the candidate segments that are specific for B and C species hexon proteins. All of the screened epitopes were finally mapped to the 3D structure of each model with Rasmol 1.74 [51] software. The RET analysis and candidate epitope screening process were performed using programs written in Bioperl script language on a Dell PowerEdge 2900 workstation.

## Results

### Phylogenetic analysis

Phylogenetic analysis is critical for determining the progressive modeling path and calculating the site-homology in RET analysis. To accurately determine the evolutionary distances among the thirteen hexon family proteins and the template sequences and to determine the modeling path, NJ method was used to construct the phylogenetic trees which were constructed based on a distance matrix [37]. A smaller distance indicates a closer evolutionary relationship. Figure 1 shows that the phylogenetic tree can be divided into two branches, a branch of the B species and a branch of the C. Although 2OBE is a chimpanzee adenovirus hexon and 1P2Z and 1P30 are human adenovirus hexons, based on the distance relationships, 2OBE is genetically closer to the nine B species human adenovirus hexon proteins than 1P30 and 1P2Z. This result is consistent with previous reports [24], [26]; therefore, 2OBE formed the template for modeling the B species hexons and 1P2Z and 1P30 formed the templates for modeling the C species hexons. It should be noted that the evolutionary relationship between the B1 (B3, B7, B16, B21, and B50) and B2 (B11, B14, B34, and B35) is not separated clearly, thus the difference between B1 and B2 is not located in the hexon gene.

### Homology modeling and molecular dynamics simulation of B16, C2, and C5

**Figure 2:**
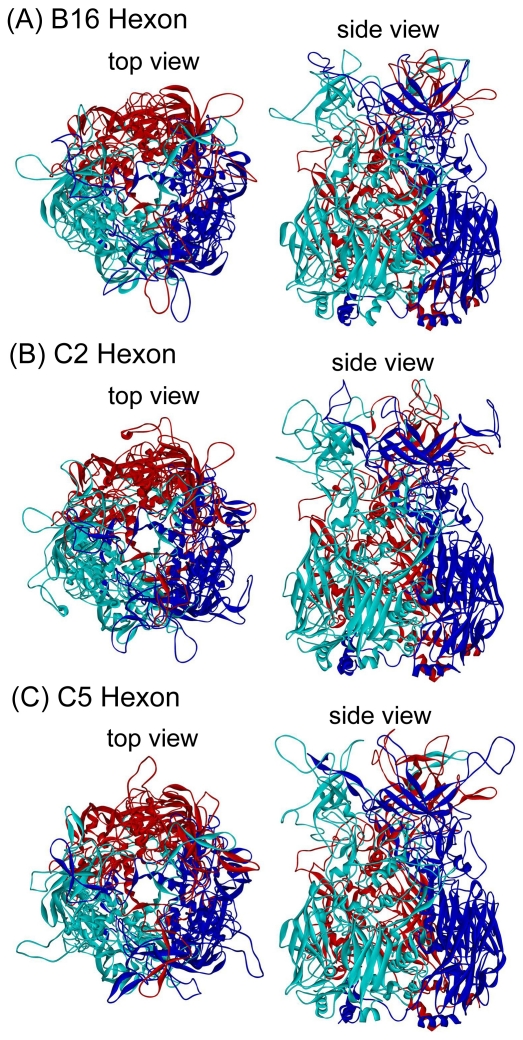
The secondary structures of HAdV B16, C2, and C5 hexons. The representative secondary structure of B16(A), C2(B), and C5(C) hexons is shown with top view (left) and side view (right), with the random coil, sheet, helix, and turn represented. Three monomers are displayed in red, blue, and cyan, respectively.

Only C2 and C5 structures have been resolved (1P2Z and 1P30) thus far [15], [24]. For the B16, C2, and C5, the authors utilized traditional homology modeling methods to obtain their initial structures. The complete human adenovirus hexon is a homotrimer containing a total of about 2800 amino acids. The Modeler program was used to build the 3D structure of B16, C2, and C5 hexon homotrimer and a total-10 ns MD simulation was performed to refine the initial structures. Figure 2 shows that the B16, C2, and C5 hexon homotrimers all form stable structures with three interlinked hexon monomers, which is consistent with our previous results and the findings from other studies [24], [25], [26]. The base region contains many sheet and helix structures, which are important to maintain the stability of the hexon protein. Three tower regions (including Loop1 and Loop2) sit on top of the trimer. Each of these tower regions forms part of the two monomers by cross-section. The number of turns and random coils increases remarkably in these regions, especially in the exposed regions on the surface of the hexon. Therefore, according to the characteristics of B cell epitopes, these turns and coils may contain the epitopes.

**Figure 3:**
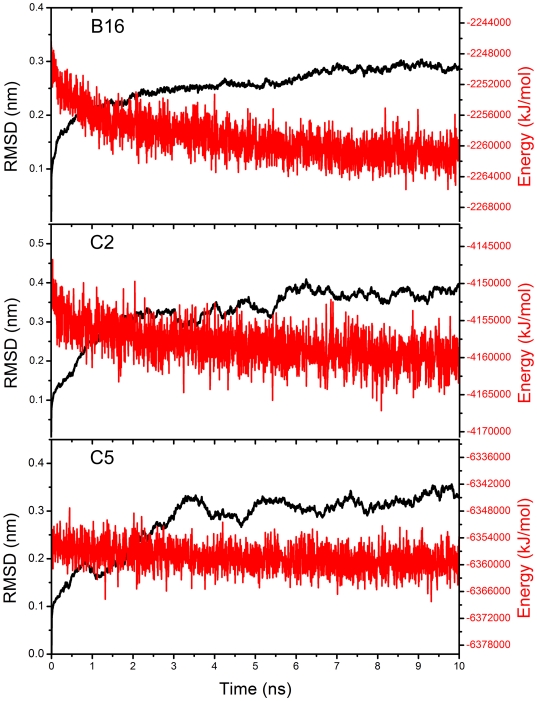
Analysis of MD simulations through potential energy and RMSD. The potential energy and the RMSD of B16, C2, and C5 hexons in 10-ns MD simulation are shown on red and black lines respectively. The blue dashed frames contain the balanced stages of three MD simulations.

B16, C2, and C5 were used as the starting point of the modeling path. Because the quality of their structures directly determines the modeling of other downstream hexons, it is essential to refine them thoroughly and precisely to obtain the closet and most reasonable 3D structures. Moreover, MM cannot solve the problem of energy barriers. After MM of the initial theoretically calculated structure of three structures, it was necessary to perform a long-range MD simulation in an explicit water solvent environment to obtain a stable and reasonable final conformation of B16, C2, and C5 hexon. As shown in Figure 3, when the simulation lasted for a certain time (B16 was approximately 7 ns; C2 6 ns; and C5 3 ns), PE and RMSD reached a balance.

**Figure 4:**
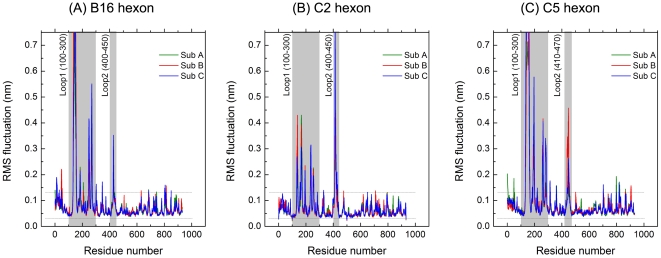
RMSF for B16, C2, and C5 hexon trimer in the final balanced stage of MD simulations. The RMSF plots for three hexon subunit monomers of B16 (A), C2 (B), and C5 (C) with green (Monomer A), red (Monomer B), and blue lines (Monomer C). The flexibility of most of the amino acid residues fluctuated less than 1 Å (between the two dotted lines), indicating that in equilibrium, three hexons were very stable, whereas the epitope-containing Loop 1 and 2 region showed greater flexibility even in the final balanced stage.

RMSF is the standard deviation of atomic displacement. RMSF of Cα atoms is a residue-based property defined over a certain time to reflect the differences in residue mobility within and between simulations. RMSF was calculated over the final balancing stage of each MD simulation. Figure 4 shows the RMSF per residue of three hexon proteins over the final balancing stage of the MD simulation. The residues in Loop 1 and 2 showed the greatest flexibility, and the secondary structure of both loops mainly comprised turns and random coils. This finding indicates that the conservative core structure of hexon has little flexibility, and this stable structural feature ensures the stability of the whole viral capsid, whereas the residues in Loop 1 and 2 display great flexibility, expand into the outside space and exhibit immunogenicity. Together, these data suggest that Loop 1 and 2 contain the epitopes.

### Rapid modeling and structure assessment

A higher degree of similarity between homologous sequences reduces the computational need for modeling of hexon family proteins while keeping as much accuracy as possible. Based on the relationship among the thirteen hexon proteins in this study, an optimized modeling path was designed and carried out on the Accelrys Discovery Studio 2.5 platform to build the thirteen models. In this study, two methods were used to evaluate the protein structures. Profile-3D was used to assess the compatibility of an amino acid sequence with a known 3D protein structure, and the Procheck program was used to verify that the model displayed reasonable stereochemistry.

**Figure 5:**
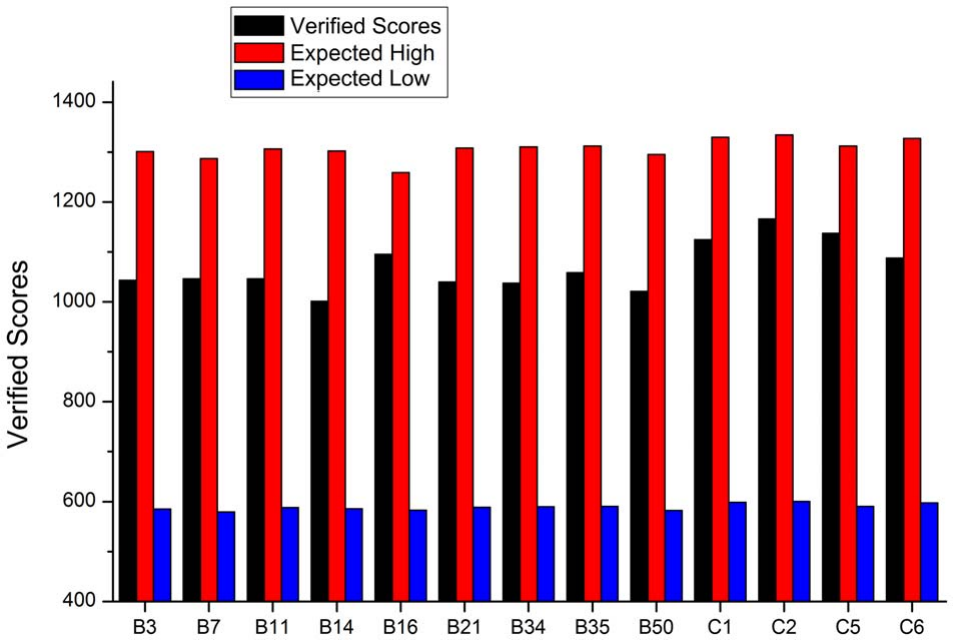
Profile-3D verification of reasonable folding in the structures of thirteen hexons. The verified scores, expected high and expected low scores of 13 hexon proteins are shown in black red and blue columns. If the overall quality score is close or higher than the expected high score, some or all of the structure is reasonable. If the overall quality is close or lower than the expected low score, then the structure maybe misfolded and needs to be further remodeled or refined.

Profiles-3D evaluates the fitness of a protein sequence in its current 3D environment. It can be applied to assess the quality of a theoretical model or to examine the characteristics of an experimental structure. The Verify Score of a protein is the sum of the scores of all residues in the protein. As shown in Figure 5, all 13 hexons have received good Verify Scores, close to the expected high score, much higher than the expected low score, suggesting that these hexon structures are acceptable.

**Figure 6:**
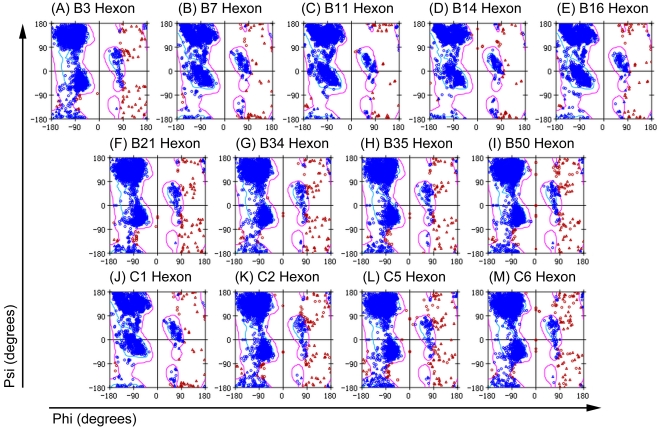
Ramachandran plots of thirteen hexon structures. The Ramachandran plot shows phi-psi torsion angles of all residues in the structure. The coloring/shading on the plot represents the different regions: the darkest areas correspond to the “core” regions representing the most favorable combinations of phi-psi values. The percentage of residues in the “core” regions is one of the better indications of stereochemical quality. The Ramachandran plots demonstrate that approximately 75% of the backbone amino acid residues (ϕ) and dihedral angles (ψ) are in the central areas, and approximately 24% are in allowed regions. Only approximately 1% are in disallowed regions in the thirteen hexon models. Similar results were observed for the original template 2OBE.

The Procheck program was employed in this study to evaluate 3D chemical parameters (Figure 6). More than 95% of the main chain ϕ residues and ψ dihedral angles in each model were in the core area, and only 1% were in the untrusted zone. These findings are similar to those in the crystal structure of the template 2OBE, indicating that all thirteen models have good stereochemical features, and the hexon proteins created by the rapid modeling method are acceptable. A brief summary of the Procheck assessment is shown in Table 2.

**Table 2:** Structural assessment of thirteen hexon proteins by Procheck.

	Favored (%)	Additional (%)	General (%)	Disallowed (%)
B3	82.8	15.1	1.4	0.7
B7	83.5	15.4	1.2	0.7
B11	82.1	15.8	1.2	0.9
B14	80.7	16.7	1.6	1.0
B16	82.9	15.7	0.9	0.6
B21	80.9	16.7	1.8	0.7
B34	82.0	15.5	1.6	0.9
B35	82.1	15.7	1.0	1.2
B50	82.0	15.7	1.5	0.8
C1	87.8	10.7	1.4	0.1
C2	86.3	12.7	0.9	0.1
C5	90.3	8.9	0.7	0.1
C6	88.4	10.5	0.9	0.2

### Comparison to the traditional homology modeling method

The new modeling method is based on the high homology among the protein family members. High homology replaces a large amount of modeling calculation and refinement, and total calculation is greatly reduced. To test the reliability of the results from the new method, the authors have also used the traditional method of homology modeling to model the other 10 target sequences (not including B16, C2, and C5 hexons). The modeled structures were compared with the structures from the new method, and the results show that the RMSD of 10 models from traditional method and rapid methods is 0.093, 0.081, 0.080, 0.074, 0.111, 0.078, 0.123, 0.098, 0.083, and 0.096 nm respectively. Generally when RMSD value of two superimposed structures is less than 0.1 nm, the two structures are considered as the same; therefore, we conclude that the new modeling method is a fast and reliable protein prediction method for protein family and the resulted structures can be used as the basis for subsequent analysis.

**Figure 7:**
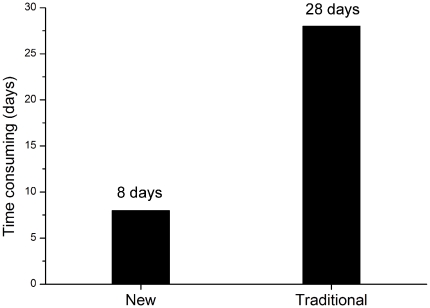
Time comparison between the new and the traditional modeling methods. The black column shows the time (days) consumed by the new modeling method and the traditional homology modeling working with the 13 hexons from B and C species HAdVs.

The time frame of the two methods was compared only considering the same computing environment ignoring other unimportant complex factors, and the CPU time occupied by the two methods (in hour units) was measured. Distribution of the molecule numbers in different procedure of the two methods was different as shown in Table 3. The initial structure modeling by Discovery Studio platform (DS modeling) is the same between the two methods and the difference lies in the molecular refinement. For the new rapid modeling method there are 10 molecules refined in Discovery Studio platform (DS refinement) and only 3 in Gromacs refienment, but for the traditional one, all 13 molecules are refined by Gromacs MD simulation. The initial model of each hexon protein modeling (DS modeling) was generally completed in 2 hours (Dell PowerEdge 2900 workstation), and the DS refinement of each molecule was about 4 hours (Dell PowerEdge 2900 workstation). Each molecule solvent MD simulation for 10 ns performed by Gromacs (Gromacs refinement) took about 48 hours (DAWNING Supercomputer Center). As shown in Figure 7, the new modeling approach for these 13 molecular took about 8 days (13×2+10×4+3×48 = 190 hours), but the traditional modeling method has taken about 28 days (13×2+0×4+13×48 = 650 hours) to complete the analysis.

**Table 3:** Distribution of the number of molecules in different procedure of two modeling methods.

	DS modeling	DS refinement	Gromacs refinement
New	13	10	3
Traditional	13	13	0

### High-throughput epitope analysis

Not all serotype-specific HVRs and not all segments on the surface of a molecule are epitopes. Only those that are located on the molecular surface and are serotype-specific are potential epitopes. We had developed a simple hexon epitope screening algorithm combining the data from SAS and the site homology analysis of RET according to the two features of hexon serotype-specific B cell epitopes. The present study has improved this method by adding a second screening calculation cycle and a second MSA to refine the serotype-specific segments in B and C species hexon proteins.

This algorithm combines structural features with bioinformatics analysis based on two important features of the serotype-specific B cell epitopes of HAdV hexon proteins: 1) all epitopes are B cell epitopes and are located on the surface of the hexon homotrimer and 2) the epitopes of HAdV hexon proteins are all serotype-specific. Importantly, these epitopes were defined as being serotype-specific within B and C species HAdVs. These structural and bioinformatics features were implemented in the hexon epitope screening algorithm using the Bioperl programming language. A summary of candidate epitope peptides from the thirteen hexon proteins is shown in Table 4. Serotype B3, B16, C2, C5 and C6 hexons present five specific B cell epitope peptides, serotype B7, B14, B50 and C1 present four epitope peptides, and the rest the hexon proteins only present three epitope peptides.

**Table 4:** The identified candidate epitopes of thirteen hexon proteins from HAdV B and C species.

Serotype	Location	Sequence	Location	Sequence
B3	S1: (135–142)	NGDNAVTT	S2: (169–175)	TTTEGEE
	S3: (240–250)	KPTTEGGVETE	S4: (263–270)	DAVAGALA
	S5: (419–435)	VKTDDTNGWEKDANVAP		
B7	S1: (135–140)	GEDNAT	S2: (234–243)	TPTEGDVEAE
	S3: (256–262)	EAADAFS	S4: (412–426)	PRDTAWEKDTKVSTA
B11	S1: (135–150)	KNTTGEEHVTEEETN	S2: (178–183)	VSDEES
	S3: (428–437)	NAPNWKEPEV		
B14	S1: (136–151)	ETTEERQNEDGENDEK	S2: (178–184)	VPAEGDP
	S3: (245–252)	VKTEEAGN	S4: (427–434)	QAWKDVNP
B16	S1: (131–138)	KDSDSKMH	S2: (162–171)	IDSTSGTDTV
	S3: (229–241)	NLKDSETAATTPN	S4: (251–261)	NKNIAANYDPD
	S5: (413–429)	AVAGTSGTQWDKDDTTV		
B21	S1: (136–151)	KKEDGGSDEEEEKNLT	S2: (177–184)	SEITDGEA
	S3: (429–438)	QGADWKEPDI		
B34	S1: (136–153)	TSTGLVDDGNDDDGEEAK	S2: (247–258)	VKPKEDDGTNNI
	S3: (432–440)	QSTWTNVDP		
B35	S1: (136–153)	PTAAAAGNGEEEHETEEK	S2: (181–187)	ISAENES
	S3: (432–441)	DNNNWKEPEV		
B50	S1: (132–144)	NKGDEEDGEDDQQ	S2: (171–177)	VPSEGGP
	S3: (238–244)	VKKEEEG	S4: (420–430)	ETTTWKDLEPK
C1	S1: (133–144)	EEPTQEMAEELE	S2: (158–167)	AEAPQADQKV
	S3: (194–199)	TQTEGN	S4: (275–284)	APSGTAMNER
C2	S1: (133–142)	TEDSGRAVAE	S2: (157–165)	EQNARDQAT
	S3: (192–198)	NAETQAK	S4: (275–284)	NTTSLNDRQG
	S5: (443–448)	SGDNGD		
C5	S1: (130–140)	DEAATALEINL	S2: (146–158)	DNEDEVDEQAEQQ
	S3: (182–188)	VEGQTPK	S4: (264–275)	TEATAGNGDNLT
	S5: (420–432)	LTKVKPKTGQENG		
C6	S1: (133–144)	NETAQVDAQELD	S2: (150–162)	ANEAQAREQEQA
	S3: (186–198)	TADATVAGAGKEI	S4: (273–283)	STNATNEVNNI
	S5: (434–443)	TTAANGDQGN		

**Figure 8:**
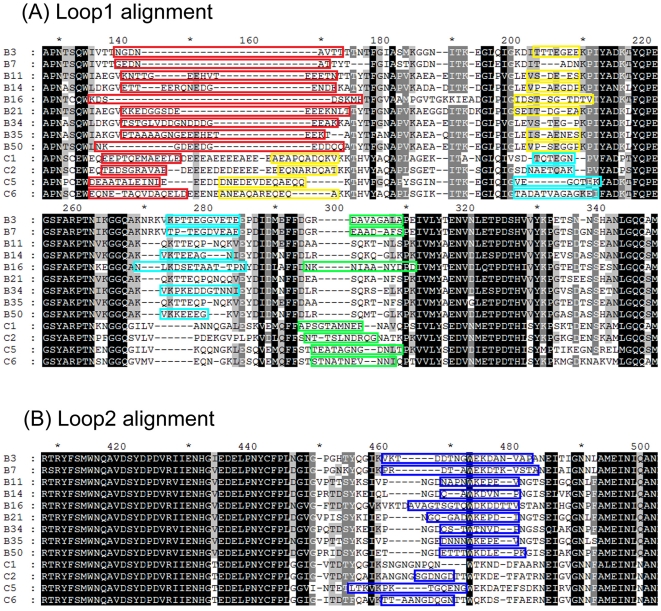
Multiple sequence alignment of thirteen hexon proteins from B species HAdVs. Boxes colored with red, yellow, cyan, and green in Loop1 (A) and blue in Loop2 (B) show the epitope peptides in the primary sequences. Serotype B3, B16, C2, C5, and C6 hexons present five specific B cell epitope peptides; serotype B7, B14, B50, and C1 present four epitope peptides; and the rest hexon proteins only present three epitope peptides.

**Figure 9:**
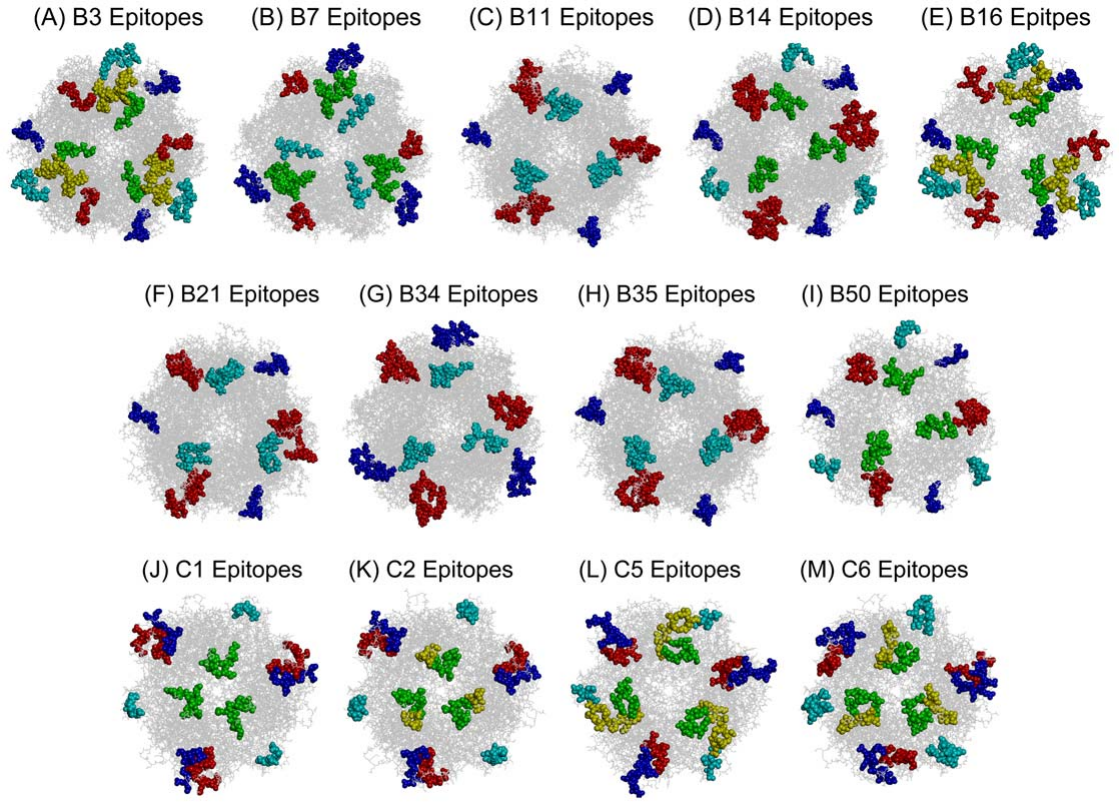
Mapping the identified epitopes onto the final 3D structures of the hexon proteins from B and C species HAdVs (top view). (A–M): Pictures A–I show the epitope mapping on the hexon proteins of HAdV species B3, B7, B11, B14, B16, B21, B34, B35, B50, C1, C2, C5, and C6. The epitopes are indicated by spacefill representation and colored with same color scheme used in Figure 8.

All the epitope peptides located on the aligned MSA sequences in the Loop1 and Loop2 regions are shown in Figure 8. There were at least seven HVRs on the hexon proteins, but only the serotype-specific peptides on the surface of the protein were recognized as serotype-specific B cell epitopes during epitope screening. The candidate epitopes were mapped onto the built 3D structures (Figure 9). The results indicate that all serotype-specific epitope candidates of hexon proteins were located in the top tower regions and exposed on the outside surface, but the number, location, and size of epitopes differed among there viral serotypes.

## Discussion

The traditional typing methods for infectious HAdVs are mostly based on virus isolation and culture or PCR reaction, which are complex and time consuming and cannot meet the requirement for rapid typing of HAdV infection. The identification of type-specific epitopes on hexons can provide necessary information for the development of rapid diagnostic typing kit. Our previous study has established a homology modeling-based epitope mapping method, but how to model a family of multiple proteins as the HAdV hexons for high-throughput epitope analysis remains a key issue for developing rapid HAdV typing reagents.

Increasing numbers of families of proteins have been sequenced, but the speed of growth of technology for protein structure determination has not been as fast as that for sequencing. For example, HAdV hexon superfamily (consisting 54 proteins) has been sequenced by high-throughput genome sequencing technology [14]; however, only HAdV2 and HAdV5 hexon protein structures have been determined by X-ray crystal diffraction technology and deposited in the PDB.

Hexon protein is a homotrimer containing nearly 2,800 amino acids. Using the traditional homology modeling method to analyze the numerous hexon protein sequences identified through high-throughput genome sequencing technology is cumbersome and requires a huge amount of computation. The conventional homology modeling technology is currently facing tremendous pressures from two recent developments. First, with the rapid development of systems biology driven by the advent of whole-genome sequencing technology, a growing number of biological macromolecules have been sequenced. Some of these biological macromolecules and polymers may contain hundreds, thousands, or hundreds of thousands of atoms. The traditional method of homology modeling and structure refinement for deciphering the structures of these macromolecules cannot meet the associated exponential growth in computational needs. These computational needs are far beyond the capacity of the linear growth in the capability of modern computers; therefore, traditional homology modeling is not suitable for routine laboratory use. Second, among the newly discovered proteins, there are a large number of protein superfamilies, many of which contain numerous family members. For the structural modeling of large numbers of family proteins, the traditional one-by-one modeling strategy becomes operationally tedious and time consuming, and the results are often unreliable due to excessive computing steps.

Here, we have reported a novel rapid molecular modeling method based on the phylogenetic tree of protein family sequences. The new method requires less computation and is easy to perform. To make the results of rapid modeling more reliable, in addition to the basic rules, two more criteria should be noted: the homology value among protein family members must be high, and the distance-based phylogenetic tree of protein family members must be constructed accurately, i.e., the MSA must be performed precisely to determine the optimal modeling path. Higher homology values between target sequences and template sequences indicate a shorter distance between them in their evolutionary relationship as well as less computation required for homology modeling and structure refinement, which is considered as the “first principle of homology modeling.” According to this principle, this study constructed a phylogenetic tree based on the distance between thirteen B and C species HAdV hexons using the NJ method. Using the information provided in the phylogenetic tree, a progressive modeling path was determined to reduce the computational need for rapid homology modeling and structural refinement. The rapid modeling data in this study are reliable and applicable to the modeling of the other ∼40 serotypes of HAdV hexons or to other similar highly homologous protein families.

This study has also used the traditional methods to remodel and refine all molecules, and compared the results from both methods. The time taken to complete the analysis was also compared. The results indicate that the new method predicts almost the same structures compared to the traditional method but consumes much less computing resources. The new modeling method reduces the huge amount of computation by taking advantage of the high homology among protein family members to achieve both accuracy and high reliability of modeling results.

The RET method and the hexon epitope screening algorithm developed in our previous study were improved in this work. Using these improved methods, serotype-specific hexon epitopes were identified in thirteen B and C species HAdVs. The results show that all the candidate epitopes are located in the top tower region of hexons and are fully exposed to the outer surface, with the secondary structure composed mainly by random coils, impling that during the long term of co-evolution of the adenoviruses and the hosts, hexon evolves and changes, but the phenotypic changes in hexon are concentrated in its tower region, which forms the structural basis for the current 54 hexon serotypes. All serotypes of HAdV hexons, including some non-human adenoviral hexons, share an almost identical conservative core, the structure of which is crucial for maintaining the survival of the virus. This structurally conserved core is located at the base of the hexon. In contrast, primary sequences located closer to the tower region share less homology among serotypes. In addition, the peptide sequences located on the top of tower are completely exposed to the outside and thus present serotype specificity in site-homology calculation.

Although the candidate epitopes identified in this study are theoretically specific for each HAdV serotype and meet the general criteria for a B cell epitope, these computationally predicted epitopes require further experimental verification. Moreover, the epitope screening algorithm is not absolute and has room for further optimization. For example, identification of the secondary structure can be included as one of the criteria, and the MD simulation of epitope peptides can be used to analyze the dynamic characteristics of candidate epitopes. Altogether, the novel epitope screening strategy described in this study has overcome the blindness of the traditional method for B cell epitope prediction and provided a new direction to the field.

Notably, the epitopes in these thirteen hexons differ in both quantity and location. It is generally believed that HAdV hexon proteins contain a number of serotype-specific HVRs that are located at different sites in the hexon homotrimer; however, HVRs are not equivalent to epitopes. Using a purposely designed hexon epitope screening algorithm, this study has shown that B3, B16, C2, C5, and C6 hexons present five specific B cell epitope peptides; serotype B7, B14, B50, and C1 present four epitope peptides, and the rest of the hexon proteins only present three epitope peptides. This indicates that the composition of epitopes in different hexon serotypes is complex, and the number, location, size, and 3D structure of these epitopes vary greatly.

It should be noted that our epitope analysis is clearly goal-oriented in nature. The type-specific epitopes identified can be used directly for development of rapid diagnostic reagents for adenovirus typing. These epitopes are not the total epitopes on hexon proteins. For example, B35 has only three type-specific epitopes identified, but in fact at least two other peptide segments can immunize the body to produce corresponding specific antibodies, though they are not type-specific epitopes. They share almost the same primary sequences and 3D structures with B11 and other serotypes. Therefore, the epitope analysis in this study is to be used for the development of diagnostic reagents and not suitable for the use to study total immunological response and epitope composition of hexons or to instruct the construction of recombinant adenoviral vectors for gene therapy.

This study has also analyzed the RMSF of B16, C2, and C5 hexons over the final balanced stage. The RMSF of Cα atoms is a residue-based property defined over a certain period of time that reflects the differences in residue mobility within and between simulations. The result of RMSF indicates that even in the final balanced stage, two epitope-containing loops (Loop 1 and 2) in B16, C2, and C5 show greater flexibility and float continuously outside rather than forming a stable, clustered conformational epitope. This finding provides preliminary evidence that the hexon epitope composition is at least partially linear rather than completely conformational. However due to the complexity of hexon epitope composition, the extent to which the hexon epitope composition is linear or conformational remains controversial.

In summary, this study has developed a novel rapid method for the modeling of homologous protein molecules based on their phylogenetic tree. This method takes advantage of the high degree of homology among the members of protein families to minimize the computational needs for homology modeling and structure refinement. In order to ensure accuracy, the structure of the node molecule B16, C2, and C5 were deeply refined to prevent expansion of structural errors along the modeling path. Subsequently, based on RET analysis, an improved hexon serotype-specific B cell epitope screening algorithm was adopted to screen all candidate epitopes based on two major criteria: the structural characteristics of B cell epitopes and the specificity of epitopes for B and C species HAdVs. Finally, serotype-specific candidate B cell epitopes were successfully identified in B and C species hexon proteins. The results also indicate that the size, location, and 3D structure of the predicted epitopes differ among the thirteen serotypes. The compositions of epitopes in different hexon serotypes are complex and await further investigation. The present work provides a theoretical basis for the future development of rapid HAdV diagnostic agents and establishment of a complete HAdV epitope database.
